# Prevalence, antimicrobial resistance, and enterotoxin gene profiles of *Staphylococcus aureus* isolated from mobile phones of the food vendors in Phayao province, Thailand

**DOI:** 10.1186/s12941-023-00621-y

**Published:** 2023-08-07

**Authors:** Krissana Khoothiam, Watsawan Prapasawat, Atchariya Yosboonruang, Anchalee Rawangkan, Chorpaka Phuangsri, Kitwadee Rupprom, Parinya Kraivuttinun, Wimonrat Tanomsridachchai, Orasa Suthienkul, Achiraya Siriphap

**Affiliations:** 1 Division of Microbiology, School of Medical Sciences, University of Phayao, Phayao, Thailand; 2 Department of Clinic, Faculty of Veterinary Medicine, Mahanakorn University of Technology, Bangkok, Thailand; 3 Department of Clinical Pathology, Faculty of Medicine Vajira Hospital, Navamindradhiraj University, Bangkok, Thailand; 4 Department of Environmental Science, Faculty of Science and Technology, Uttaradit Rajabhat University, Uttaradit, Thailand; 5 Faculty of Public Health, Mahidol University, Bangkok, 10400 Thailand

**Keywords:** *Staphylococcus aureus*, Mobile phone, Food vendor, Antimicrobial resistance, Staphylococcal enterotoxin

## Abstract

**Background:**

Mobile phones are widely used and may cause bacterial pathogens to spread among various professionals. *Staphylococcus aureus* from the mobile phones can contaminate the hands of food vendors and food during the cooking or packaging process. This research aimed to determine the prevalence, enterotoxin genes, and antimicrobial resistance (AMR) profiles of *S. aureus* contaminating the vendors’ mobile phones.

**Methods:**

In this study, 266 mobile phone samples were randomly collected from food vendors selling food on walking streets (n = 139) and in food centers (n = 127) in Phayao province. All samples were identified as *S. aureus* by the conventional culture method and confirmed species-specific gene by polymerase chain reaction (PCR). Then, all identified *S. aureus* isolates were tested for antimicrobial susceptibility by broth microdilution method and for the presence of staphylococcal enterotoxin (SE) genes by PCR.

**Results:**

The results showed that 12.8% of the mobile phones collected were contaminated with *S. aureus*. Of 49 *S. aureus* isolates obtained, 30 (61.2%) were positive for SE genes. The most common SE gene was *sea* followed by *sec*, *seb*, *sem*, *seq*, and *sel*. Moreover, *S. aureus* was most frequently resistant to penicillin, followed by chloramphenicol and tetracycline, erythromycin, clindamycin, and gentamicin. Methicillin-resistant *S. aureus* (MRSA), vancomycin-resistant *S. aureus* (VRSA), and multidrug*-*resistant (MDR) strains were also detected.

**Conclusions:**

This study showed that mobile phones were an intermediate surface for the transmission of *S. aureus*, including MDR variants. It indicates that hand hygiene and the decontamination of mobile phones are essential to prevent cross-contamination of *S. aureus* in food settings.

## Introduction

Mobile phones have become one of the most important accessories in both professional and social life. Although they are handy and convenient for communication, mobile phones may pose a health risk due to the presence of thousands of microorganisms on their surface [1]. Many previous reports have revealed that mobile phones may be contaminated with pathogenic bacteria and nosocomial pathogens including *Staphylococcus aureus* and MRSA [2]. Pathogenic bacteria, including MDR strains, have been detected on the mobile phones of hospital personnel in many countries, with *S. aureus* being the most common, followed by MRSA, *S*. *epidermidis,* and other Gram-negative pathogens [2]. Antimicrobial-resistant *S. aureus,* including MRSA, has also been reported to contaminate the mobile phones of medical personnel in Thailand [3]. Previous studies indicated that *S. aureus* may play a significant role in causing food poisoning outbreaks [4, 22].

*S. aureus* is a pathogenic bacterium and major cause of food poisoning worldwide, including in Thailand [5, 22]. *S. aureus* present in food can multiply and produce enterotoxins. The consumption of foods containing staphylococcal enterotoxins (SEs) may cause food poisoning. Although *S. aureus* is killed by heat during cooking, SEs are heat-stable and not easily destroyed. There are 23 types of SEs and staphylococcal-like enterotoxins (SE-like toxins). Five important serological types are SEA, SEB, SEC, SED, and SEE [6]. SEA was the main enterotoxin causing food poisoning. Moreover, SEA coexisting with SEG was the most frequently found enterotoxin in retail ready-to-eat foods [6].

Various ready-to-eat foods sold on walking streets and in food centers have become increasingly popular in Thailand. At the same time, almost all food vendors increasingly use their mobile phones in the food business. Therefore, mobile phones may serve as the perfect surface for the transmission of microorganisms, especially those from the human surface membrane. *S. aureus* from nose or skin infections can be spread to and survive on the surface of mobile phones via the hands and eventually be transferred into food. This may become a health risk, causing food poisoning or other illnesses to consumers.

There are no current published reports on the presence of *S. aureus* carrying SEs on the mobile phones of food vendors. Generally, several antimicrobial agents are used to treat *S. aureus* and MRSA infections, except for food poisoning [7]. Antimicrobial-resistant strains, especially MRSA, VRSA, and MDR, have been reported to cause nosocomial infections [8]. However, to our knowledge, no studies have been published to date on the contamination of food through mobile handheld devices with foodborne pathogens like *S. aureus* in food vendors’ settings in Thailand and elsewhere. Therefore, this study aimed to determine the prevalence of *S. aureus* and to investigate MDR strain contamination on the mobile phones of food vendors. The enterotoxin gene profile of *S. aureus* was also examined.

## Methods

### Sample collection

A total of 266 mobile phone samples were randomly collected from each food vendors on walking streets and in food centers (wet markets, 24-h markets, and canteens) in Phayao province, Thailand, from January to March 2021. The samples were collected by rubbing sterile cotton swabs soaked with sterile phosphate-buffered saline (PBS) over the screens of the mobile phones and then placing them in peptone water (PW). All PW samples were incubated at 37 °C for 24 h after arrival at the laboratory.

### Isolation and identification of *S. aureus*

A loopful of the inoculated PW culture was subsequently streaked on mannitol salt agar (MSA; HiMedia Laboratories Pvt. Ltd., Mumbai, India) and Baird Parker agar (BPA; HiMedia Laboratories Pvt. Ltd., Mumbai, India) supplemented with egg yolk tellurite emulsion. After incubating at 37 °C for 24–48 h, typical colonies of *S. aureus* were sub-cultured on tryptic soy agar (TSA; HiMedia Laboratories Pvt. Ltd., Mumbai, India) and blood agar (BA; HiMedia Laboratories Pvt. Ltd., Mumbai, India). The production of beta-hemolysis on BA indicated the presence of *S. aureus* [9]. The colonies from TSA showing Gram-positive reaction with grape-like clusters were further identified by biochemical tests (oxidase, catalase, and coagulase tests) and confirmed by species-specific gene (*fem*A) detection using PCR [10].

### Antimicrobial susceptibility test

Antimicrobial susceptibility to 19 antimicrobial agents, namely ampicillin (AMP), penicillin (PEN), oxacillin (OXA), vancomycin (VAN), teicoplanin (TEC), daptomycin (DAP), gentamicin (GEN), erythromycin (ERY), tetracycline (TET), levofloxacin (LVX), moxifloxacin (MXF), ciprofloxacin (CIP), clindamycin (CLI), trimethoprim/sulfamethoxazole (SXT), rifampin (RIF), chloramphenicol (CHL), cefotaxime (CTX), linezolid (LZD), and tigecycline (TGC), was tested by broth microdilution method with Sensititre THAPF following the Clinical and Laboratory Standards Institute (CLSI) guidelines. The antimicrobial susceptibility results were interpreted according to CLSI breakpoints (CLSI, 2021) except for tigecycline, for which the clinical breakpoint according to the European Committee on Antimicrobial Susceptibility Testing (EUCAST) recommendations was used. *S. aureus* ATCC 25923 was used as a reference strain. In this study, intermediate or resistance to more than three antimicrobial classes was defined as MDR [11].

### DNA extraction

Genomic DNA (gDNA) of *S. aureus* was extracted using the DNeasy Blood and Tissue Kit (Qiagen, Hilden, Germany) as described in the manufacturer’s instructions. The DNA was stored at − 20 °C until use.

### PCR assay

The gDNA of the oxacillin-resistant *S. aureus* isolate (MIC ≥ 4 µg/ml) was used to determine the presence of methicillin resistance genes (*mecA* and *mecC*) by PCR to confirm the MRSA strain, as previously described by Stegger et al. [12]. Subsequently, all *S. aureus* isolates were subjected to the detection of nine SE genes (*sea*, *seb*, *sec*, *sed*, *sej*, *ser*, *sem*, *sel*, and *seq*) by PCR using specific primers [13, 14]. The oligonucleotide primers, PCR product size, and annealing temperature are described in Table 1 [15, 16]. PCR amplifications were performed in a total volume of 25 μl according to OnePCR™ Ultra (GeneDireX, Inc., Taiwan). Each reaction consisted of 12.5 μl of OnePCR™ Ultra (Taq buffer, MgCl_2_, dNTPs, and *Taq* polymerase; GeneDireX, Inc., Taiwan), 0.5 μl of each primer (10 μM), 10.5 µl of distilled water, and 1 μl of DNA template. The PCR protocol was slightly modified according to OnePCR™ Ultra (GeneDireX, Inc., Taiwan) and performed in a PTC-100 Thermocycler (MJ Research Inc., Watertown, MA, USA). Briefly, the amplification conditions were as follows: initial denaturation step at 94 °C for 5 min followed by 35 cycles of denaturation at 94 °C for 30 s, annealing for 1 min (temperatures are shown in Table 1), extension at 72 °C for 2 min, and final extension step at 72 °C for 5 min. The PCR products were analyzed by 1.5% (w/v) agarose gel electrophoresis. The desired PCR products were purified and commercially sequenced by Macrogen Inc. in South Korea. The DNA similarity was performed with GenBank. The sequences were submitted to GenBank, with accession numbers ON109381 to ON109386.

**Table 1:** PCR primers used in this study

Gene	Primer	Oligonucleotide sequence (5'-3')	Annealing temperature (^o^C)	Amplicon size (bp)	References
*femA*	*femA*-F	TACGCAGCATATACCGCACT	54	300	[10]
	*femA*-R	CCATTACTGGACCACGATTC			
*mecA*	*mecA*-1	AAAATCGATGGTAAAGGTTGGC	51	533	[15]
	*mecA*-2	AGTTCTGCAGTACCGGATTTGC			
*mecC*	*mecC*-1	GCTCCTAATGCTAATGCA	51	304	[16]
	*mecC*-2	TAAGCAATAATGACTACC			
*sea*	*sea-*1	ACGATCAATTTTTACAGC	44.5	544	[13]
	*sea-*2	TGCATGTTTTCAGAGTTAATC			
*seb*	*seb-*1	ATTCTATTAAGGACACTAAGTTAGGGGA	44.5	404	[13]
	*seb-*2	ATCCCGTTTCATAAGGCGAGT			
*sec*	*sec*-1	GACATAAAAGCTAGGAATTT	44.5	257	[13]
	*sec*-2	AAATCGGATTAACATTATCCA			
*sed*	*sed*-1	CAAATATATTGATATAATGA	44.5	330	[13]
	*sed*-2	AGTAAAAAAGAGTAATGCAA			
*sej*	*sej*-F	CACCAGAACTGTTGTTCTGCTAG	55	114	[14]
	*sej*-R	CTGAATTTTACCATCAAAGGTAC			
*ser*	*ser*-F	TCCCATTCCTTATTTAGAATACA	52	440	[14]
	*ser*-R	GGATATTCCAAACACATCTGAC			
*sem*	*sem*-F	AGTTTGTGTAAGAAGTCAAGTGTAGA	52	178	[14]
	*sem*-R	ATCTTTAAATTCAGCAGATATTCCATCTAA			
*sel*	*sei*-F	TGGACATAACGGCACTAAAA	52	145	[14]
	*sei*-R	TTGGTARCCCATCATCTCCT			
*seq*	*seq*-F	ATACCTATTAATCTCTGGGTCAATG	52	222	[14]
	*seq*-R	AATGGAAAGTAATTTTTCCTTTG			

### Statistical analysis

Descriptive statistics were used to determine the prevalence and frequency of *S. aureus* carrying enterotoxin genes and resistant strains. Data were analyzed using the Chi-square or Fisher’s exact tests with the statistical package SPSS (Version 21.0) and Microsoft Excel 2013. The *p-*value < 0.05 was considered statistically significant.

## Results

### Prevalence of *Staphylococcus aureus* on mobile phones

In this study, a total of 266 mobile phones were swabbed for sample collection from food vendors in Phayao province. The prevalence of *S. aureus* detected on mobile phones was 12.8% (34/266). The contamination of *S. aureus* on the mobile phones of food vendors on walking streets (11.5%; 16/139) and in food centers (14.2%; 18/127) was not significantly different (*p* > 0.05).

### Staphylococcal enterotoxin genes

All 49 *S. aureus* isolates obtained from the 34 positive samples were tested for nine SE genes by PCR. The results indicated that the detection rate of SE genes was 61.2% (30/49; Table 2). The most frequent SE gene was *sea* at 32.7% (16/49), followed by *sec* (20.4%; 10/49), *seb* (10.2%; 5/49), *sem* (8.2%; 4/49), *seq* (4.1%; 2/49), and *sel* at 2.0% (1/49). However, *sed*, *sej*, and *ser* were not detected. Additionally, *S. aureus* carrying two SE genes (*sea*, 43.5% and sec, 13.0%) was detected in the samples from walking streets, while six genes (*sec*, 26.9%; *sea*, 23.1%; *seb*, 19.2%; *sem*, 15.4%; *seq*, 7.7%; and *sel*, 3.8%) were detected in samples from food centers. However, the frequency of SE genes from food centers (65.4%; 17/26) was not significantly higher than that of SE genes from walking streets (56.5%; 13/23;* p* > 0.05).

**Table 2:** Distribution of *Staphylococcus aureus* isolates carrying staphylococcal enterotoxin genes from mobile phones of food vendors

Place	No. of isolate	No. (%) of *S. aureus* carried *se* gene	No. (%) of *S. aureus* carried								
			*sea*	seb*	*sec*	*sed*	*sej*	*sem*	*sel*	*seq*	*ser*
Walking street	23	13 (56.5)	10 (43.5)	0	3 (13.0)	0	0	0	0	0	0
Food center	26	17 (65.4)	6 (23.1)	5 (19.2)	7 (26.9)	0	0	4 (15.4)	1 (3.8)	2 (7.7)	0
Total	49	30 (61.2)	16 (32.7)	5 (10.2)	10 (20.4)	0	0	4 (8.2)	1 (2.0)	2 (4.1)	0

^*^p-value < 0.05

Additionally, 30 *S. aureus* isolates carried one to three SE genes that were grouped into eight SE gene profiles as follows: *sea* (40.0%; 12/30), *seb* (10.0%; 3/30), *sec* (23.3%; 7/30), *seq* (3.3%; 1/30), *sea*-*sec* (6.7%; 2/30), *sea*-*sem* (6.7%; 2/30), *seb*-*sem* (6.7%; 2/30), and *sec*-*sel*-*seq* (3.3%; 1/30; Table 3). Food centers were found to contain all eight SE gene profiles (*sec* [4/17], *seb* [3/17], *sea* [2/17], *seq* [1/17], *sea-sec* [2/17], *sea-sem* [2/17], *seb-sem* [2/17], and *sec-sel-seq* [1/17]), while walking streets had only two profiles (*sea* [10/13] and *sec* [3/13]).

**Table 3:** Antimicrobial resistance and enterotoxin gene profiles of *Staphylococcus aureus* isolates

Antimicrobial resistance profiles^*^	No. of isolates	No. of isolates carried SE genes	No. of isolates staphylococcal enterotoxin gene profiles							
			*sea*	*seb*	*sec*	*seq*	*sea-sec*	*sea-sem*	*seb-sem*	*sec-sel-seq*
CHI	5	4	1	–	1	–	–	2	–	–
PEN	4	1	–	–	1	–	–	–	–	–
TET	1	0	–	–	-	–	–	–	–	–
ERY-CHL	2	1	1	–	-	–	–	–	–	–
PEN-CHL	2	1	–	–	1	–	–	–	–	–
PEN-DAP	1	1	1	–	–	–	–	–	–	–
PEN-TET	6	4	3	–	–	–	–	–	1	–
TET-CHL	1	1	–	1	–	–	–	–	–	–
PEN-CLI-CHL	1	1	–	–	–	–	–	–	–	1
PEN-ERY-CHL	1	1	–	–	–	1	–	–	–	–
PEN-TET-CHL	5	5	4	–	1	-	–	–	–	–
PEN-TET-ERY	3	0	–	–	–	-	–	–	–	–
PEN-DAP-TET-CLI	1	1	–	1	–	-	–	–	–	–
PEN-MXF-ERY-CHL	1	0	–	–	–	-	–	–	–	–
PEN-TET-SXT-CHI	1	1	1	–	–	-	–	–	–	–
PEN-TET-SXT-CIP	1	0	–	–	–	-	–	–	–	–
DAP-TET-GEN-ERY-CHL	1	1	–	–	–	-	1	–	–	–
PEN-MXF-ERY-CLI-CHL	1	0	–	–	–	-	–	–	–	–
PEN-OXA-GEN-ERY-CLI-CHL	1	1	–	–	1	–	–	–	–	–
PEN-OXA-GEN-ERY-RIF-CLI	1	1	–	–	1	–	–	–	–	–
PEN-OXA-TET-SXT-GEN-CLI	1	0	–	–	–	–	–	–	–	–
PEN-TET-SXT-ERY-CLI-CHI	1	0	–	–	–	–	–	–	–	–
PEN-OXA-TET-GEN-RIF-CLI-CHL^a^	1	1	–	1	–	–	–	–	–	–
PEN-OXA-DAP-TET-GEN-ERY-RIF-CLI	1	1	–	–	–	–	–	–	1	–
PEN-MXF-DAP-TET-GEN-CIP-LVX-CLI	1	1	1	–	–	–	–	–	–	–
PEN-OXA-DAP-GEN-RIF-LVX-RIF-CLI-CHL	1	1	–	–	1	–	–	–	–	–
VAN-PEN-OXA-DAP-LZD-ERY-TEC-RIF-CLI-CHL^a^	1	0	–	–	–	–	–	–	–	–
Susceptible	2	1	–	–	–	–	1	–	–	–
Total	49	30	12	3	7	1	2	2	2	1

^*^PEN Penicillin, *CHL* Chloramphenicol, *TET* Tetracycline, *ERY* Erythromycin, *CLI* Clindamycin, *GEN* Gentamicin, *OXA* Oxacillin, *DAP* Daptomycin, *RIF* Rifampin, *SXT* Trimethoprim/sulfamethoxazole, *MXF* Moxifloxacin, *CIP* Ciprofloxacin, *LVX* Levofloxacin, *VAN* Vancomycin, *LZD* Linezolid, *TEC* Teicoplanin, *TGC* Tigecycline

^a^*mecA* gene positive by PCR

### Antimicrobial resistance of *Staphylococcus aureus*

**Fig. 1:**
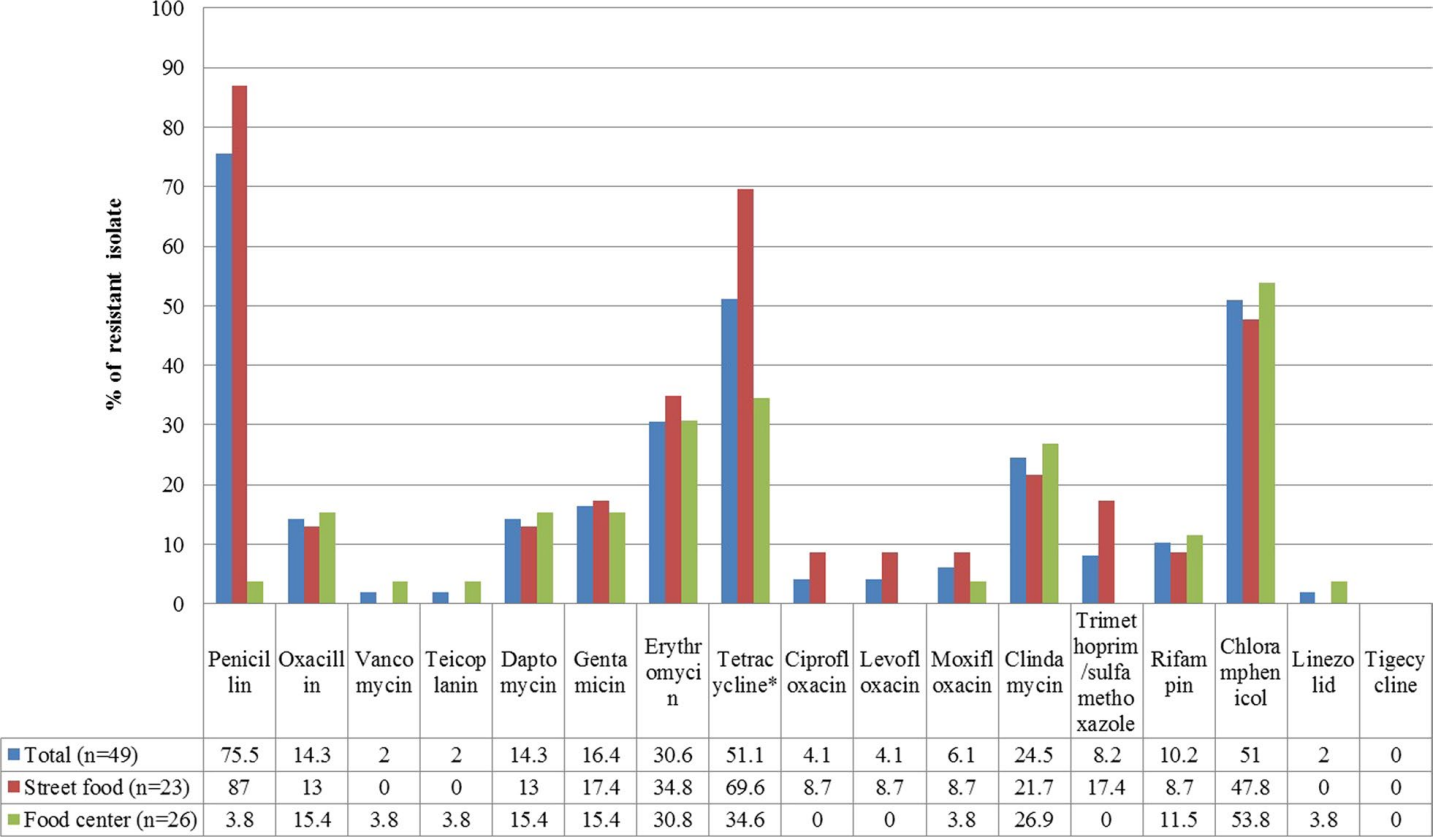
Antimicrobial resistance of *Staphylococcus aureus* isolated from mobile phones of food vendors

Antibiotic resistance profiles were determined for 17 antimicrobial agents belonging to 14 classes by the broth microdilution method. The AMR of *S. aureus* isolates is shown in Fig. 1. In this study, 95.9% (47/49) of all isolates were resistant to at least one antimicrobial agent. The frequency of AMR was as follows: PEN (75.5%), followed by CHL and TET (51.0% each), ERY (30.6%), CLI (24.5%), GEN (16.3%), OXA and DAF (14.3% each), RIF (10.2%), SXT (8.2%), MXF (6.1%), CIP (4.1%), and LVX, VAN, LZD, and TEC (2.0% each).

In this study, only the frequency of TET resistance of *S. aureus* isolates detected on mobile phones from walking streets (69.6%; 16/23) and food centers (34.6%; 9/26) was significantly different (*p* < 0.05). All isolates were susceptible to TGC.

Furthermore, only one isolate (2.1%) from a food center (a wet market) exhibited vancomycin resistance (MIC > 32 µg/ml) and was defined as a VRSA strain. Additionally, 2/7 (28.6%) OXA-resistant (MIC > 4 µg/ml) isolates harboring *mecA* were defined as MRSA and found from a walking street and a food center. Furthermore, MDR was found in 25 isolates (51.0%; 25/49), 64.0% (16/25) from walking streets and 36.0% (9/25) from food centers. In this study, 27 AMR profiles of *S. aureus* isolates were found, as shown in Table 3. The common AMR profile in MDR isolates was PEN-TET-CHL (20%; 5/25); all such isolates were found on walking streets. Moreover, interestingly, an MRSA (*mecA* +) strain having the PEN-OXA-TET-GEN-RIF-CLI-CHL resistance profile carrying *seb* was found in a food shop at a food center. Additionally, another MRSA (*mecA* +) strain having the VAN-PEN-OXA-DAP-IZD-ERY-TET-RIF-CLI-CHL resistance profile but not carrying any SE genes was found at the food center. Conversely, 1/2 susceptible strains carrying *sea-sec* was detected at the food center.

## Discussion

The use of mobile phones provides many advantages; however, it may be a source of pathogen contamination, such as with *S. aureus* [1]*. S. aureus* has emerged as a major pathogen for both hospital and community-acquired infections. It can contaminate food or material products during preparation and processing. *S. aureus* can survive in dry and stressful environments such as the nose, skin, clothing, and surfaces [17]. These characteristics support the growth of *S. aureus* in many food products [11]. In addition, *S. aureus* can remain viable on hands and environmental surfaces for a long time after contact [18]. Hands may contaminate mobile phones at the time of use, especially when it is hot and humid and the hands are sweaty [19]. For all these reasons, there is no doubt that *S. aureus* is possibly present on the mobile phones of people in various occupations, such as healthcare workers, hospital staff, medical students [20], university students [21], and the food vendors. However, no study has previously assessed the contamination of *S. aureus* on the mobile phones of food vendors. Our study showed that 12.8% of mobile phones were contaminated with *S. aureus* among the ready-to-eat food vendors; this is within the prevalence range reported for previously studied samples from phones belonging to those in other occupations. This result also agrees with a study where *S. aureus* was detected in ready-to-eat food samples in northeast Thailand [6], where the prevalence was 37.7%.

Accordingly, while mobile phones offer many of the advantages to food vendors, including (1) the better quality and flexibility of service offered to customers, (2) the ability to accept payments wirelessly, (3) increased ability to communicate in and out of the workplace, (4) greater access to modern apps and services, and (5) improved networking capabilities, it is of a concern that they may be a conduit for the transmission of potentially pathogenic organisms. When comparing mobile phone contamination by *S. aureus* among food vendors either on walking streets (11.5%) or in food centers (14.2%), the prevalence in these two settings was not significantly different (*p* > 0.05). It is concluded that the mobile phone is one of the potential vehicles for *S. aureus* dissemination into food during food preparation regardless of the size of the food shop.

*S. aureus* enterotoxin is the major cause of food poisoning and other public health problems in developing countries [22]. In Thailand, many episodes of foodborne disease outbreaks have occurred without investigation of the causative agents due to (1) not being able to immediately collect the relevant food samples and (2) a much higher incidence as sporadic cases of *S. aureus* infection are not adequately reported. It is only known that *S. aureus* is the third most common causative agent of foodborne illness in Thailand [23]. Conversely, several studies have assessed the detection rates of SEs in food samples such as ready-to-eat foods [24] and retail chicken meat [25]. In this study, SE genes were detected in 61.2% (30/49) of all isolates from samples collected from the mobile phones of food vendors. The *sea* gene was found at a higher frequency than others. The present results agreed with several previous studies reporting that *sea* was the most common gene in *S. aureus* isolated from food [24–26]. However, *sed*, *sej*, and *ser* were not detected in our study, which did not agree with a previous study reporting the presence of these genes in food poisoning cases and food. Additionally, *sed*, *sej*, and *ser* are known to be located on plasmids. In our study, eight *se* genotypes were observed and 23.4% of isolates possessed more than one SE gene: *sea*-*sec* (6.7%), *sea*-*sem* (6.7%), *seb*-*sem* (6.7%), and *sec*-*sel*-*seq* (3.3%). However, the onset of *S. aureus*-mediated food poisoning is abrupt. Abdominal cramps, nausea, and vomiting are the most common symptoms but the infection is generally self-limiting and resolves within 24–48 h. The conclusive diagnostic criteria of *S. aureus* food poisoning are based on the detection of SEs in food or exposure to at least 10^5^ cell/g from food [27]. Therefore, the toxigenic *S. aureus* detected on mobile phones should be further analyzed for the phenotypes of toxin production and also detected in the relevant food samples for food safety.

The growing problem of AMR is a major public health concern. Although most studies of AMR surveillance have focused on healthcare and agriculture settings, AMR in humans and environments has also been reported. Staphylococci are commonly found in built environments. Multiple studies have indicated that AMR bacteria, including *S. aureus,* can be transmitted to humans in public environments including on buses [28], at railway stations [29], and in classrooms [30]. Presently, much evidence of AMR *S. aureus* contaminating mobile phones has been derived from healthcare settings where it causes nosocomial infection; *S. aureus* resistant to ampicillin, oxacillin, ceftazidime, vancomycin, and amoxicillin has been isolated from the mobile devices of students in the health sector [31]. Additionally, *S. aureus* isolates resistant to ceftazidime (50%), gentamycin (40.9%), ciprofloxacin (40.9%), tetracycline (36.4%), chloramphenicol (31.8%), imipenem (27.3%), and azithromycin (27.3%) were isolated from the mobile phones of healthcare workers in Bangladesh [2]. However, there is no known surveillance of AMR *S. aureus* on the mobile phones of food vendors. Only *S. aureus* isolates resistant to erythromycin, ciprofloxacin, oxacillin, and cefoxitin were detected in processed raw meat/fish samples of ready-to-eat foods in other settings [32]. This study’s results regarding the AMR of *S. aureus* on the mobile phones of food vendors were similar to those of previous reports in other settings. It is remarkable that the frequency of penicillin resistance was high, at 75%, and that 28.6% of isolates harbored *mecA*. These variants detected on the mobile phones of food vendors on walking streets and in food centers, were therefore defined as MRSA.

Furthermore, our data agreed with those from ready-to-eat foods, humans, pork, and beef [32]. Conversely, the prevalence of MDR *S. aureus* was quite high (51.0%) in this study. Most of the MDR isolates were methicillin-sensitive *S. aureus* and diverse. Only MRSA and VRSA isolates having MDR profiles of PEN-OXA-TET-GEN-RIF-CLI-CHL and VAN-PEN-OXA-DAP-LZD-ERY-TEC-RIF-CLI-CHL were detected in this study. It seemed that the AMR profiles in this study were quite different from those in previous studies [31]. This may be due to the antimicrobial agents used in different settings and the different environments, times, or samples. Thus, the results of one study may not be comparable with those of other studies. Within the same study, the AMR of *S. aureus* from the mobile phones of food vendors on walking streets and in food centers was quite similar. Only the percent resistance to each drug was different but not so significantly. However, MRSA isolate having MDR profile and carrying *seb* was detected on a mobile phone from a food shop at a food center in this study. It is not known whether this isolate was derived from food vendors, food materials, or other related environments in the shop. Generally, food is also an important factor in the transfer of AMR. Recently, MRSA strains were isolated from several food-producing animals including pigs, cattle, chickens, and other animals [27]. Additionally, this strain could produce enterotoxin when exposed to optimal conditions, leading to food poisoning outbreaks. It is concluded that the diversity of the *S. aureus* population on the mobile phones of food vendors regarding their toxigenic potential and AMR sheds light on the quality and safety of ready-to-eat foods on walking streets and in food centers.

## Conclusions

To our knowledge, this is the first report to study the mobile phones of food vendors both on walking streets and in food center shops for the presence of *S. aureus*. Contamination by MDR *S. aureus* strains, including MRSA carrying SE genes, was detected on the mobile phones of food vendors. Thus, the mobile phone of the food vendor might be carriers to spread the antimicrobial-resistant *S. aureus* strain producing SEs into the food that cause food poisoning when ingested in contaminated food. The possibility of mobile phone contamination occurring during business practices indicates the potential threat of mobile phones spreading infections, and the importance of both mobile phone hygiene and hand hygiene to prevent infection must be emphasized.

## Data Availability

All data generated or analyzed during this study are included in this published article.
